# Canadian Children and Youth in Care: The Cost of Fetal Alcohol Spectrum Disorder

**DOI:** 10.1007/s10566-013-9226-x

**Published:** 2013-08-20

**Authors:** Svetlana Popova, Shannon Lange, Larry Burd, Jürgen Rehm

**Affiliations:** 1 Social and Epidemiological Research Department, Centre for Addiction and Mental Health (CAMH), 33 Russell Street, Room # T507, Toronto, ON M5S 2S1 Canada; 7 Social and Epidemiological Research Department, Centre for Addiction and Mental Health (CAMH), Toronto, ON Canada; 2 Dalla Lana School of Public Health, University of Toronto, Toronto, ON Canada; 3 Factor-Inwentash Faculty of Social Work, University of Toronto, Toronto, ON Canada; 4 Institute of Medical Sciences, University of Toronto, Toronto, ON Canada; 5 Department of Pediatrics, University of North Dakota School of Medicine, Grand Forks, ND USA; 6 Epidemiological Research Unit, Klinische Psychologie and Psychotherapie, Technische Universität Dresden, Dresden, Germany

**Keywords:** Fetal alcohol syndrome, Fetal alcohol spectrum disorder, Children/youth in care, Child welfare, Cost, Canada

## Abstract

**Background:**

A high prevalence of prenatal alcohol exposure has been reported among children in care and thus, the risk of fetal alcohol spectrum disorder (FASD) in this population is high.

**Objective:**

The purpose of the current study was to estimate the number of children (0–18 years) in care with FASD and to determine the associated cost by age group, gender, and province/territory in Canada in 2011.

**Methods:**

The prevalence of children in care by province/territory was obtained from the Canadian Child Welfare Research Portal, and the number of children in care with FASD for each province/territory was estimated from available epidemiological studies. In order to calculate the total cost per province/territory, the cost per individual per day, by age group, was applied to the respective number of children in care with FASD.

**Results:**

The estimated number of children in care with FASD ranged from 2,225 to 7,620, with an annual cost of care ranging from $57.9 to $198.3 million Canadian dollars (CND). The highest overall cost ($29.5 to $101.1 million CND) was for 11–15 year-olds.

**Conclusion:**

The study findings can be used to demonstrate the substantial economic burden that FASD places on the child welfare system. Attention towards the needs of this population and prevention efforts to reduce FASD incidence in Canada, and other countries are urgently needed.

## Introduction

Children and youth^1^ in care represent a unique population with disproportionately increased rates of developmental disabilities, congenital malformations, mental health diagnoses, and social maladjustment (Chernoff et al. 1994; Fuchs et al. 2008; Harman et al. 2000; Hostetter et al. 1991; Lindblad et al. 2003).

Children who are placed in care often are due to a number of unfavourable circumstances, such as, parental and/or drug problems, child abuse and/or neglect, child abandonment, and young maternal age. Such circumstances are likely to increase the likelihood that a child was exposed to alcohol in utero (Burd et al. 2011; Herrick et al. 2011). Thus, the risk of fetal alcohol spectrum disorder (FASD) in this population is likely to be high. FASD is not a diagnostic term, but is an umbrella term encompassing four categorical diagnostic entities: fetal alcohol syndrome (FAS), partial FAS, alcohol-related neurodevelopmental disorder, and alcohol-related birth defects (Chudley et al. 2005; Stratton et al. 1996). FAS is the most severe and visibly identifiable form of FASD. Prenatal alcohol exposure can affect any organ or system of the fetus, therefore, individuals with FASD may have a broad array of physical defects, cognitive, behavioural, emotional, and adaptive functioning deficits, as well as congenital anomalies, such as malformations and dysplasia of the cardiac, skeletal, renal, ocular, auditory, and other systems. These impairments are likely to have lifelong implications.

In a recently conducted study by the authors of this article, utilizing the current epidemiological and medical literature, over 300 disease conditions coded in the International Classification of Diseases, version 10 were identified to occur in individuals with FASD (Popova et al. *in progress*). The demonstrated complexity and chronicity of FASD draws attention to the fact that these affected individuals require a wide range of assistance from multiple service systems, including health care, community organizations, remedial education, and others. Without crucial support, people affected by FASD are at an increased risk of developing secondary disabilities, such as mental health problems, trouble with the law, school drop-outs, unemployment, homelessness, and/or alcohol and other drug problems (Streissguth et al. 2004). When combined with the child’s primary deficits, these secondary disabilities increase the complexity of care and result in significant social and economic costs to society (Abel and Sokol 1987; Harwood 2000; Legge et al. 2001; Lupton et al. 2004; PHAC 2003, 2005; Popova et al. 2011b, 2012b; Stade et al. 2009).

In the general population of Canada, the crude prevalence of FAS and FASD have been reported as 1 per 1,000 (Roberts and Nanson 2000) and 9 per 1,000 (PHAC 2003), respectively. However, the prevalence of FASD among children in care^2^ has been reported to be much higher. For example, in the province of Manitoba, of the 1,869 children in care identified as having a disability, 640 (34 %) had FASD and an additional 280 (15 %) were suspected to have FASD (Fuchs et al. 2005). Based on a total of 5,664 children in care in Manitoba at this time, the prevalence of FASD within this population can be estimated to be 113 per 1,000, which is about 13 times higher than the estimated prevalence of FASD among the general population.

A recent analysis, conducted by the authors of this paper, revealed an alarming prevalence of FASD among children in care in different countries (Lange et al. 2012b, 2013). For example, in Chile, the prevalence of FAS and FASD among children (1 to 20+ years of age) in the care of child welfare (“protective”) services and homes for those with mental deficiencies was reported as 62 per 1,000 and 158 per 1,000, respectively (Mena et al. 1993). In the USA, the prevalence of FAS among children, up to the age of 13, living in foster care was found to be 10–15 per 1,000 (Astley et al. 2002). Further, the prevalence of FAS in foster homes and orphanages in Russia was reported to be extremely high-150 per 1,000 (Bubnov 2010)—and in orphanages for children with special needs, the prevalence was even higher—ranging from 427–680 per 1,000 (Legon’kova 2011). In Brazil, the prevalence of FASD among children residing in orphanages was reported as 277 per 1,000 (Strömland et al. 2011).

The severity of FASD and its associated disabilities are a significant and direct predictor of removing children from their birth homes and placing them in foster care. Thus, Kvigne et al. (2004) reported that among their sample of Northern Plains American Indian children, the children with full-blown FAS were 64 times more likely to be removed from their homes and children with partial FAS were 14 times more likely to be removed from their homes, when compared to children without FAS or partial FAS. Further, children with full-blown FAS were 28 times more likely to be placed in foster care and 14 times more likely to be living with their relatives and children with partial FAS were 5 times more likely to be placed in foster care and twice as likely to be living with relatives, when compared to children without FAS or partial FAS.

There are only a few studies in Canada and the USA that have attempted to estimate the cost associated with FASD (no studies exist in any other country; Popova et al. 2011b, c, 2012b). In the existing FASD-cost analyses (Abel and Sokol 1987, 1991a, b; Harwood 2000, 2003; Harwood et al. 1984, 1998; Harwood and Napolitano 1985; Rice et al. 1990, 1991; Rice 1993; Weeks 1989), the cost of children in care with FASD was not included in the overall estimates. In these studies, the total cost associated with FASD might be underestimated based on the fact that children with FASD are overrepresented in child care systems (Farris-Manning and Zandstra 2003; Hutson 2006; Popova et al. 2012c, d).

There are only a couple of studies in Canada that have estimated the cost of children in care with FASD. Fuchs et al. (2008) estimated that in 2006 the total annual cost of 400 children in care with FASD in Manitoba, one Canadian province, was $9.5 million. The authors found that the average total daily cost of caring for a child with FASD in the child welfare system was $65 (or $23,760 per annum). However, a study by Stade et al. (2009) estimated that the average annual direct cost per child with FASD in foster care is about $2,000 in Canada. The noticeable disparity between the costs reported by two existing studies is likely due to the utilization of different methodologies and the inclusion and exclusion of different cost components.

The current study is part of a large economic project, with multiple components, working towards estimating the overall burden and cost associated with FASD in Canada (Lange et al. 2012a; Popova et al. 2011a, 2012a, c, d, 2013a, b). Until now, an estimation of the number of children in care with FASD and the associated cost, at the national level, has not been undertaken in Canada, or in any other country.

### Hypotheses

It was hypothesized, based on the current Canadian and international literature, that the prevalence of children in care with FASD is likely higher than in the general population and thus, the economic cost associated with children in care with FASD in Canada is considerable.

### Study Objectives

The current study was designed to: (1) estimate the number of children in care with FASD by age group, gender, and province/territory and (2) estimate the associated cost of children in care with FASD in Canada in 2011.

An estimation of the cost of FASD for the Canadian child welfare system is central to describing the extent of its impact on this population, the cost to society, and for evaluating the potential benefits of FASD prevention programs. Furthermore, the current economic estimate has the potential to provide additional policy insights in order to better address the needs of this unique population and to increase awareness of this problem not only in Canada, but also internationally.

## Method

### Design

The current study was a modeling study using secondary data.

### Ethics Statement

Given that the current study utilized secondary data reported on the aggregate level, which is readily available in the literature, it was not necessary to obtain research ethics approval.

### The Total Number and Prevalence of Children in Care in Canada

The latest estimates of the total number of children (0–18 years of age) in care and the prevalence estimates of children (0–18 years of age) in care per 1,000 among the general population in Canada, by province/territory in 2007 were obtained from the Canadian Child Welfare Research Portal (http://www.cecw-cepb.ca/statistics; CRCF 2011; Table 1). The methodology of deriving a national estimate of children in care by province/territory is described in a report by Mulcahy and Trocmé (2010). This study used multiple ascertainment strategies to compile available statistics presented in federal, provincial, and territorial documents and websites, including: (a) placement statistics compiled by Human Resources and Social Development Canada for the Federal/Provincial/Territorial Directors of Child Welfare Committee for years 1992–2004 and Social Security statistics on the number of children in out-of-home care from 1971 to 2003; (b) information from Indian and Northern Affairs Canada tracking on reserve Aboriginal children in out-of-home care from 1969 to 2007; (c) statistics reported by provincial and territorial authorities in their annual reports and/or their websites; and (d) statistics reported by provincial/territorial associations (e.g., provincial/territorial Children’s Aid Societies), or through provincial/territorial reviews/reports. The national estimates of children in care by province/territory were calculated using Statistics Canada population estimates for the year 2007 for children 0–18 years of age (Mulcahy and Trocmé 2010).

**Table 1:** The number of children (0–18) in the general population and in care, as well as the prevalence of children in care per 1,000 by province/territory in Canada, 2007 (CRCF 2011)

Province/territory	Number of children in the general population	Number of children in care	Prevalence of children in care in the general population per 1,000
Alberta	841,392	8,891	10.6
British Columbia	915,168	9,271	10.1
Manitoba	297,004	7,241	24.4
New Brunswick	154,395	1,388	9.0
Newfoundland and Labrador	102,857	768	7.5
Northwest Territories	12,810	395	30.8
Nova Scotia	194,389	1,706	8.8
Nunavut^a^	12,839	197	15.3
Ontario	2,931,745	18,763	6.4
Prince Edward Island^b^	31,713	166	5.2
Quebec	1,625,581	12,750	7.8
Saskatchewan	251,271	5,447	21.7
Yukon	7,212	178	24.7
Canada	7,378,376	67,161	n/a

*n/a* not available

^a^Nunavut data are from 2006 because no data on children in care were available for 2007

^b^Prince Edward Island data are a monthly count

### The Cost of Caring for Children in Care with FASD in Canada

The cost of care per day, by age group and gender, for children in care was obtained from Fuchs et al. (2008). These cost figures were for 2006, but in the current study are reported as inflation adjusted costs for 2011, using the inflation calculator of the Bank of Canada (http://www.bankofcanada.ca/rates/related/inflation-calculator/; see Table 2).

**Table 2:** The age, gender distribution, and cost of care per day for children with FASD in Canada, 2006 and 2011

Age group (years)	Age distribution (%)^a^			Cost of care ($)	
	Boys	Girls	Overall	Average cost per day for children in care with FASD in 2006^a^	Inflation adjusted average cost per day for children in care with FASD in 2011^b^
0–5	2.75	2.25	5.00	49.00	53.65
6–10	19.75	10.50	30.25	57.00	62.41
11–15	29.25	17.50	46.75	71.00	77.74
16–18	10.50	7.50	18.00	68.00	74.45

*FASD* fetal alcohol spectrum disorder

^a^Data from Fuchs et al. (2008)

^b^Adjusted for inflation, from 2006 to 2011, using the Bank of Canada inflation calculator (http://www.bankofcanada.ca/rates/related/inflation-calculator/)

The total cost of care per day, included basic maintenance, special rate/special needs, and exceptional circumstances. Basic maintenance refers to the funds that are required for the everyday costs of providing for children in care (e.g., food, utilities, child care, replacement clothing); special rate/special needs funds are those that cover costs that exceed or were not intended to be covered by basic maintenance (e.g., fees for service, therapy, medical expenses); and exceptional circumstances are funds that cover expenses that are above and beyond those required for normal care (e.g., support services, criminal legal fees, renovations required to the foster home for a disabled child).

### Data Analysis

#### Estimation of the Number of Children in Care with FASD, by Age Group, Gender, and by Province/Territory in Canada in 2011

To calculate the total number of children in care for each province/territory, the respective prevalence estimates of children in care, obtained from the Canadian Child Welfare Research Portal (CRCF 2011), were applied to the number of children in the general population for each province/territory in Canada in 2011 (obtained from Statistics Canada 2012).

There are only two estimates that exist on the prevalence of children in care with FASD for Canada. The first estimate is 33 per 1,000 (reported for the province of Ontario; Burge 2007) and the second estimate is 113 per 1,000 (reported for the province of Manitoba; Fuchs et al. 2005). In order to estimate the total number of children in care with FASD for each province/territory, these prevalence estimates were applied (as the lower and upper estimates, respectively) to the total number of children in care for each province/territory.

The age and gender distribution of children in care with FASD obtained from Fuchs et al. (2008; Table 2) and was applied to the total estimated number of children in care for each province/territory.

#### Estimation of the Cost Associated with Caring for Children in Care with FASD by Age Group, Gender, and by Province/Territory in Canada in 2011

The estimated cost figures for children in care with FASD for each age group, gender, and province/territory are reported in 2011 Canadian dollars. The cost per day (adjusted for inflation for 2011) for each age group was multiplied by 365 (number of days in the year), and applied to the estimated number of children in care with FASD.

## Results

### The Number of Children in Care with FASD, by Age Group, Gender, and by Province/Territory in Canada in 2011

#### Prevalence of Children in Care

The highest prevalence of children in care, in 2011, was in the Northwest Territories (30.8 per 1,000—1 out of every 32 children in the territory), followed by Yukon Territory (24.7 per 1,000—1 out of every 41 children) and Manitoba (24.4 per 1,000—1 out of every 41 children), as reported by the Canadian Child Welfare Research Portal (http://www.cecw-cepb.ca/statistics; CRCF 2011; Table 1). The province/territory with the lowest prevalence of children in care was Prince Edward Island (5.2 per 1,000—1 out of every 192 children in the province), followed by Ontario (6.4 per 1,000—1 out of every 156 children) and Newfoundland (7.5 per 1,000—1 out of every 133 children). However, due to differences in base population sizes, Ontario had the largest number of children in care (18,546), followed by Quebec (12,674) and Alberta (9,377). In 2011, the total number of children in care in Canada was estimated to be 67,433.

#### Estimated Number of Children in Care with FASD

The three provinces with the highest number of children in care with FASD were as follows: Ontario [612 (lower estimate) to 2,096 (upper estimate)], Quebec (418–1,432) and Alberta (309–1,060). Overall, the total number of children in care with FASD in Canada, in 2011, ranged from 2,225 to 7,620.

The estimated number of children in care (0–18 years of age) with FASD by gender, and by province/territory and the associated cost in Canada in 2011 are presented in Table 3.

**Table 3:** The number of children (0–18) in care with FASD and the associated cost by gender and by province/territory in Canada, 2011

Province/territory; gender	Number of children in the general population^a^	Prevalence of children in care in the general population^b^ (per 1,000)	Number of children in care^c^		Number of children in care with FASD			Total cost of children in care with FASD^f^ ($)	
					Lower estimate^d^		Upper estimate^e^	Lower estimate	Upper estimate
Alberta		10.6							
Boys					193		660	5,010,132.92	17,115,909.68
Girls					117		400	3,042,792.80	10,422,684.42
Total	884,645		9,377		309		1,060	8,053,925.71	27,578,594.10
British Columbia		10.1							
Boys					188		643	4,881,316.29	16,714,820.32
Girls					114		390	2,965,533.17	10,154,704.49
Total	904,568		9,136		301		1,032	7,846,849.46	26,869,514.81
Manitoba		24.4							
Boys					153		524	3,976,839.45	13,617,662.35
Girls					93		318	2,416,038.75	8,273,102.38
Total	305,052		7,443		246		841	6,392,878.20	21,890,764.73
New Brunswick		9.0							
Boys					28		95	720,339.74	2,466,617.90
Girls					17		58	437,626.10	1,498,537.85
Total	149,803		1,348		44		152	1,157,965.84	3,965,155.75
Newfoundland and Labrador		7.5							
Boys					15		52	395,633.95	1,354,746.56
Girls					9		32	240,358.45	823,045.59
Total	98,732		740		24		84	635,992.40	2,177,792.15
Northwest Territories		30.8							
Boys					8		26	198,509.09	679,743.25
Girls					5		16	120,599.70	412,962.62
Total	12,063		372		12		42	319,108.79	1,092,705.87
Nova Scotia		8.8							
Boys					33		114	868,234.25	2,973,044.55
Girls					20		69	527,476.06	1,806,205.89
Total	184,663		1,625		54		184	1,395,710.31	4,779,250.45
Nunavut		15.3							
Boys					4		14	106,874.54	365,964.34
Girls					2		9	64,929.21	222,333.35
Total	13,074		200		7		23	171,803.75	588,297.69
Ontario		6.4							
Boys					381		1,305	9,909,132.28	33,931,271.13
Girls					231		791	6,020,068.92	20,614,175.38
Total	2,897,886		18,546		612		2,096	15,929,201.19	54,545,446.51
Prince Edward Island		5.2							
Boys					3		11	87,235.55	298,715.68
Girls					2		7	52,997.98	181,477.95
Total	31,399		163		5		18	140,233.54	480,193.63
Quebec		7.8							
Boys					260		891	6,771,304.48	23,186,588.07
Girls					158		541	4,113,752.70	14,086,486.51
Total	1,624,813		12,674		418		1,432	10,885,057.18	37,273,074.59
Saskatchewan		21.7							
Boys					115		395	3,002,202.22	10,280,268.20
Girls					70		240	1,823,919.97	6,245,544.14
Total	258,944		5,619		185		635	4,826,122.19	16,525,812.34
Yukon		24.7							
Boys					4		13	100,890.16	345,472.36
Girls					2		8	61,293.53	209,883.91
Total	7,645		189		6		21	162,183.69	555,356.26
CANADA (all provinces/territories)									
Boys					1,385		4,743	36,028,644.91	123,370,814.39
Girls					840		2,877	21,888,387.33	74,951,144.48
Total	7,373,287		67,433		2,225		7,620	57,917,032.24	198,321,958.88

Due to rounding errors, columns may not add up to the totals reported

*FASD* fetal alcohol spectrum disorder

^a^Statistics Canada (2012)

^b^CRCF (2011)

^c^Estimated based on the respective provincial/territorial prevalence rates

^d^Estimated based on prevalence 33 per 1,000; Burge (2007)

^e^Estimated based on prevalence 113 per 1,000; Fuchs et al. (2005)

^f^Estimated based on the 2011 inflated cost per day; Fuchs et al. (2008)

### The Cost of Caring for Children in Care with FASD by Age Group, Gender, and by Province/Territory in Canada in 2011

The overall cost attributable to FASD among children in care ranged from $57.9 to $198.3 million (boys: $36.0 to $123.4 million; girls: $21.9 to $75.0 million) in Canada in 2011 (Table 3).

**Fig. 1:**
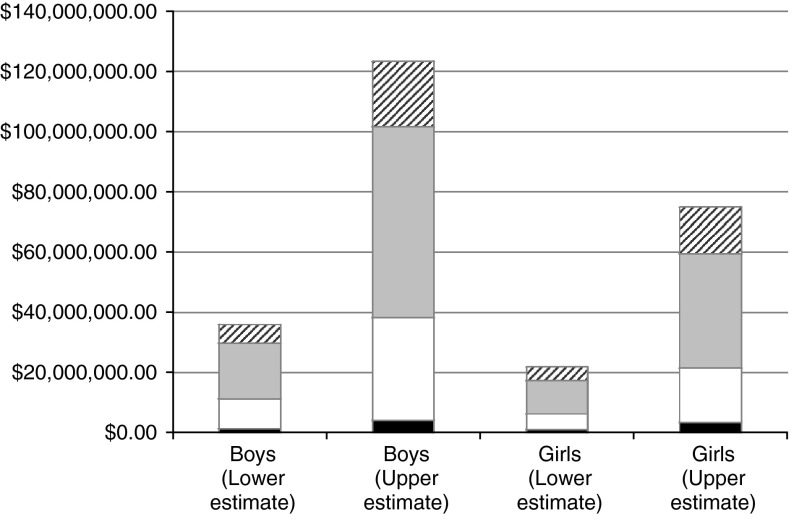
The cost of children in care with FASD by gender and age group in Canada in 2011 *striped bar* 16–18 years, *grey bar* 11–15 years, *white bar* 6–10 years, *black bar* 0–5 years

The age group associated with the highest overall cost was 11–15 years of age, followed by the age group of 6–10 years of age. The 0–5 years of age group was associated with the lowest overall cost. Please see Fig. 1 for the FASD-attributable cost of children in care with FASD by gender and age group in Canada in 2011.

## Discussion

The results of this study suggest that, in Canada, the annual cost for children in care with FASD is likely to range from $57.9 to $198.3 million. This is clearly a cost component that should not be overlooked in FASD cost studies, when considering the overall burden of FASD on any society. However, the cost of children in care with FASD, estimated in the current study, is only one component of the overall direct cost associated with FASD in Canada.

Despite the fact that the cost for children in care with FASD is substantial, it is likely that it is still underestimated for the following reasons. Firstly, raising children with FASD is a challenging undertaking, one that many foster parents may not be fully prepared for. For this reason, caregivers may choose to put a child with FASD back into provincial/territorial custody, necessitating another foster family placement, which may be associated with additional costs. Secondly, fostering children with FASD requires special considerations, such as training for both staff and foster parents, which is also likely to result in additional costs.

There are several limitations in the current study. Firstly, the estimated number of children in care in Canada in 2011 was based on the prevalence estimates reported by the Canadian Child Welfare Research Portal for the year 2007 (the most recent available year; CRCF 2011), and was assumed to remain constant during the year, which may not be accurate. Secondly, the prevalence of FASD among children in care is currently available for only two Canadian provinces–Ontario (Burge 2007) and Manitoba (Fuchs et al. 2005), which were used to estimate the prevalence of children in care with FASD in the other provinces/territories. Therefore, possible variations between the provinces/territories were not accounted for. Thirdly, due to a lack of respective provincial/territorial data, the age and gender distribution of children in care with FASD, as well as the costs of care per day, were obtained from a study based on the child welfare system in Manitoba (Fuchs et al. 2008) and applied to the other provinces/territories, which may not be generalizable to the other provinces/territories of Canada. Lastly, it should be noted that the provinces/territories of Canada vary in regard to the age at which youths are no longer permitted to be in care (i.e., some provinces/territories may only allow youths up to that age of 16 to be in care, while others may permit youths up to the age of 18 or even 21). These provincial/territorial differences were not considered in the current estimate.

Unfortunately, currently, there are no other national estimates on the cost of children in care with any other conditions/disorders. Such data would be useful to put the current estimate into context. However, it is known that the average per diem special rate/special needs cost for a child with FASD is approximately 20 % higher than that of the general children in care population ($43 versus $35; Fuchs et al. 2008). It has also been documented that children with FASD enter care at an earlier age and are more likely to become permanent wards and therefore, spend a greater proportion of the lives in care than children in care without FASD (Fuchs et al. 2008). Thus, not only are daily special rate/special needs costs of children with FASD higher, but those costs are extended over a longer period of time.

Cost studies in general have important implications for public policy. Important public policy findings of this study include: (a) funding for FASD prevention (e.g., educating women of childbearing age about the detrimental consequences of consuming alcohol while pregnant) should be a priority—if 10 % of the cost of care were devoted to prevention, Canada could allocate as much as $20 million each year to this objective; (b) improving data collection on prenatal alcohol exposure is necessary, especially for at-risk populations—this would facilitate early and accurate diagnostic evaluations; (c) enhancing access to substance abuse treatment programs for the mothers of children with FASD should be made possible—this could have substantial benefits not only for the mother and her current and future children, but also for society as a whole; and (d) increasing the effectiveness of substance abuse treatment programs for women of childbearing age, should be a principle goal—this could provide an important opportunity to prevent the occurrence and/or recurrence of FASD within families. It is a well-known fact that FASD is highly recurrent within siblings (Abel 1988). Taking a long-term perspective on this issue would suggest that increasing access to and the effectiveness of substance abuse treatment could improve the lives of several thousand children and their families (Gelb and Rutman 2011; Popova et al. 2013b).

It is also very important to identify strategies to prevent affected children from needing to be placed in care. Multiple efforts will be required to address the needs of this unique population and, via increasing the awareness, to reduce the overall prevalence/incidence of FASD in both the child care population, as well as the general population in Canada and around the world. Further, the development and implementation of programs aimed at reducing or preventing secondary disabilities, if successful, could result in very large cost savings.

The implications of this study for child care agencies include several broad areas of emphasis. Substantial rates of FASD occurrence among children in care suggest that there is an increased need for specialized training of workers, caregivers, and service providers to improve FASD recognition, for understanding the specific needs of children with FASD, and an increased need for comprehensive service plans to support children with FASD and their adoptive families. Based on the high reported prevalence of FASD in child care systems around the world (Lange et al. 2012b, 2013), children in care must be routinely screened for FASD and be referred to diagnostic services where, if necessary, a formal diagnosis can be made. Such an approach has the potential to facilitate early diagnosis, which has several noteworthy benefits (Popova et al. 2013a).

In regard to future research, the limitations of the current study draw attention to the need for accurate prevalence estimates of children in care with FASD in the provinces/territories of Canada. Also, there is a need for cost analyses to be conducted within provincial/territorial child welfare systems. Such analyses will help to make informed decisions regarding the programs, policies, and funding support for the numerous activities required to improve the lives of children in care with FASD and to prevent further alcohol-exposed births in Canada. Further, research is also needed to identify the unique medical, educational, and social needs of children in care with FASD, and their current patterns of service use (versus service needs).

The burden of children in care with FASD is not measurable by cost of care alone, the lifelong hardships faced by these children, and their families should also be considered. A strategy that could change the lives of several thousand of Canada’s most vulnerable people surely deserves consideration at this time. Prevention of FASD may well be one of Canada’s priorities for the future. For many children and their families the cost of not acting to prevent FASD and to improve the treatment of individuals with FASD may be too great to bear. If we do not act, the children in care with FASD will likely not have their needs adequately addressed, and thus, will likely experience negative outcomes, such as secondary disabilities, that will not only impact the individual, themselves, well into adulthood, but also the society in which they live (Bueller et al. 2000; Quinton et al. 1984). The substantial number of children in care with FASD, the high associated cost, and their dependence on the child care system in general, emphasizes the urgency of strategically addressing their needs. We will act, won’t we?

## Footnotes

1 The broad term “children” will be used throughout to refer to children and youth.

2 The term ‘children in care’ will be used to refer to all children in out-of-home care (i.e., those in the custody of a child welfare agency, Crown Wards [a foster child who has been made the legal responsibility of the Canadian government], and those in foster care).
